# Development of an Assessment Tool of Menstrual-Cycle-Related Signs and Symptoms Based on Thai Traditional Medicine Principles for Evaluation of Women's Health

**DOI:** 10.1155/2021/9977773

**Published:** 2021-05-21

**Authors:** Kodchanipha Sutthibut, Arunporn Itharat, Phechnoy Singchungchai, Preecha Wanichsetakul, Weerachai Pipatrattanaseree, Buncha Ooraikul, Neal M. Davies

**Affiliations:** ^1^Graduate School of Applied Thai Traditional Medicine Program, Faculty of Medicine, Thammasat University, Pathumthani 12120, Thailand; ^2^Department of Applied Thai Traditional Medicine, Faculty of Medicine, Thammasat University, Klongluang, Pathumthani 12120, Thailand; ^3^Center of Excellence on Applied Thai Traditional Medicine Researches, Faculty of Medicine, Thammasat University, Klongluang, Pathumthani 12120, Thailand; ^4^Multidisciplinary College, Christian University of Thailand, Mueang Nakhonpathom District, Nakhonpathom 73000, Thailand; ^5^Department of Obstetrics and Gynecology, Faculty of Medicine, Thammasat University, Klongluaang, Pathumthani 12120, Thailand; ^6^Regional Medical Science Center 12 Songkhla, Department of Medical Sciences, Songkhla 90100, Thailand; ^7^Department of Agricultural, Food & Nutritional Sciences, Faculty of Agricultural, Life and Environmental Sciences, University of Alberta, Edmonton AB T6G 2P5, Canada; ^8^Faculty of Pharmacy and Pharmaceutical Sciences, University of Alberta, Edmonton, Canada

## Abstract

**Background:**

Utilization of Thai traditional medicine (TTM) was considered in menstrual-cycle-related signs and symptoms (MCSs) to evaluate women's health. TTM clinicians diagnosed the MCSs by signs, symptoms, and associated factors of patients including a physical examination to find patterns of imbalance elements and the origin of the disorder to optimize treatment. Thus, the purpose of this study was to develop a new assessment tool, the menstrual-cycle-related signs and symptoms questionnaire (MCSQ) based on TTM principles for evaluation of women's menstrual health.

**Methods:**

The items and components of the MCSQ were adjusted by TTM expert consensus using the Delphi technique. The content validity of the MCSQ was quantified by the content validity index (CVI). MCSQ were examined by construct validity and internal consistency reliability using exploratory factor analysis (EFA) and Cronbach's *α* coefficient, respectively.

**Results:**

: All 19 experts (100%) responded to the questionnaires in the three rounds of the Delphi technique. The MCSQ showed high content validity of individual items (I-CVI = 0.83–1.00) and high overall content validity of the questionnaire (S-CVI/AVE = 0.98). Overall, 429 of 432 participants completed the questionnaire (99.31%). After factor analysis, the final MCSQ was divided into two sections, which consisted of 49 items. The first had 23 items focusing on the MCSs. And, the second had 14 items of personal and medical data including 12 items of associated factors. Cronbach's *α* coefficient of the final MCSQ was 0.87, and that of each component was between 0.32 and 0.82.

**Conclusions:**

This study reports a new MCS questionnaire tool, which was developed from TTM knowledge to evaluate women's health. This questionnaire showed an acceptable level of validity and reliability. Thus, it is also expected to be useful in clinical practice and ongoing research on evaluation of women's health.

## 1. Introduction

In most women, the menstruation period brings a variety of uncomfortable symptoms and inconveniences affecting daily life. Menstrual-cycle-related signs and symptoms (MCSs) are a group of physical and/or emotional manifestations that occur before or during the menstrual period, mostly identified as either premenstrual syndrome (PMS) or dysmenorrhea. These MCSs are the most prevalent menstrual problems afflicting reproductive-age women. In reproductive-age women, the prevalence of MCSs during menstruation varies between 16% and 91% [1]. The first systematic review and meta-analysis of PMS reported that the pooled prevalence of PMS worldwide was 47.8 % [2]. Although dysmenorrhea and PMS are health problems that occur in a relatively short-time period and the symptoms can disappear without treatment, both symptoms often occur every menses cycle for many years [3, 4]. Severity of symptoms directly affects the quality of life and indirectly affects social and economic development, including impaired daily living activities, and often augmented social involvement. Some adolescents and women may require absence from school or work, and may have impaired work productivity [4, 5]. Moreover, women who reported PMS at baseline were at greater risk of menopausal symptoms and risk of postpartum depression development [6, 7], and women with a history of severe dysmenorrhea symptoms before pregnancy have a higher risk of developing psychological distress during pregnancy [8]. Menstruation is an important indicator of health and quality of life in women: the reproductive endocrine system is associated with reproductive health; it affects mental health, fertility, and leads to menopause [6, 9].

Thai traditional medicine (TTM) has long been used for the treatment of menstrual disorders. According to the TTM principles, menstruation is an important part of women's health care because menstruation can be a principal cause of illnesses or diseases in women [10]. There is a specific TTM textbook about menstrual disorders and women's diseases called “Mahaa-Chotarat.” The MCSs in TTM principles are called “Lohit-Pokkathi-Thod (in Thai language).” It is characterized by symptoms and signs linked to the menstrual cycle occurring before and/or during the menstrual period and resolving before or during the onset of menstruation such as fever, low back pain, depression, irritability, characteristics of menstrual blood, and leucorrhea. This disorder is considered a dysfunction of the wind and fire elements that have an impact on water elements or effects on the blood system. Women have menstrual-cycle-related signs and symptoms; if they do not receive proper treatment, these symptoms can develop into more severe disease, such as skin disorders, mental problems, uterine abscess, or infertility and cancer [10].

The instruments and measurements are an integral component of routine patient assessment for practitioners, which ensures appropriate treatments are selected; therefore, a variety of tools for the assessment of MCSs were developed and widely used for screening or diagnostic tests such as Menstrual Distress Questionnaire (MDQ) [11], Menstrual Symptom Questionnaire (MSQ) [12], Premenstrual Symptoms Screening Tool (PSST) devised by Steiner, MacDougall and Brown 2003 [13, 14], and Calendar of Premenstrual Experiences (COPE) [15]. But, these tools focus on measuring the severity or impact of MCSs and were developed based on a conventional Western medicine context. Thai traditional medical clinicians evaluate and diagnose the MCSs by collecting signs, symptoms, and the associated behaviors of the patients. Interestingly, TTM practitioners also inspect via physical examination to diagnose for abnormalities, illness, or disease. A guided proper treatment utilizing TTM principles considers the signs and symptoms to be a reflection of the fundamental pathology of the internal systems and organs; therefore, assessing the signs and symptoms that occur leads to the fundamental pathology of the diseases. In addition, we also assess lifestyles, food and drink consumption, sleeping, mental health, and the environment to elucidate the causes of diseases, because these factors usually influence menstruation and the occurrence of MCSs according to TTM principles. Moreover, the TTM is an individualized personalized medicine approach that treats each patient on the basis of their own individual pathology. According to this foundational knowledge of TTM, we sought to determine whether it is possible to create such a tool through this study for assessment of the MCSs by applying TTM principles and knowledge.

Therefore, this study aimed to develop a menstrual-cycle-related signs and symptom questionnaire (MCSQ) based on TTM principles that can assess the associated factors, the severity of the MCSs, and identify the internal system or organ that is the cause of the MCSs. It is expected that this questionnaire and its guidelines will be clinically useful for women's self-care and prevention with precision TTM treatment.

## 2. Methods

**Figure 1:**
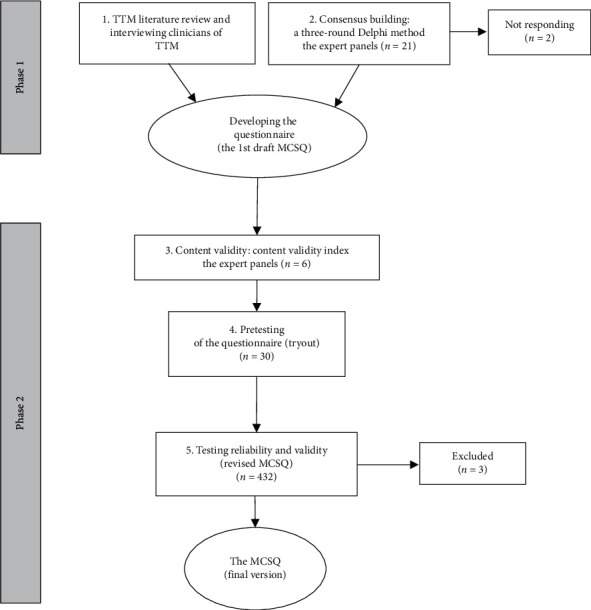
Phases and steps in developing the menstrual-cycle-related questionnaire (MCSQ).

The study employed a sequential mixed methods design consisting of two phases. The first phase was qualitative research conducted to establish the questionnaire from the literature review and interviewing TTM experts. Subsequently, the second phase was quantitative research for questionnaire development. The validity of the developed MCSQ was evaluated by using content validation and structural validation. The assessment of the reliability within each component of the questionnaire utilized internal consistency reliability. The pattern of this research is shown in Figure 1.

### 2.1. Investigation of Menstrual-Cycle-Related Signs and Symptoms Using TTM Principles

In this study, the theoretical framework about pathogenesis of MCSs was generated based on a review of the TTM literature and interviewing TTM experts–TTM clinicians who have experience in the treatment of menstrual disorders and scholars of TTM theory.

### 2.2. Developing the MCSQ Items through the Delphi Method

The Delphi technique is considered desirable to reach consensus in a field where incomplete knowledge is evident [16]. It was used to develop the appropriate MCSQ items. The Delphi method consists of repeated questionnaire surveys from experts without face-to-face meetings. It requires at least two or three rounds of the questionnaire survey if the first round is an open-ended questionnaire [17]. This study used a three-round Delphi method to establish a consensus on the essential content of assessing the cause and severity of menstrual-cycle-related symptoms based on TTM principles. The open-ended questionnaire was explored in the 1^st^ round, and the structured questionnaire was sent by mail in 2nd and 3rd rounds.

#### 2.2.1. Selection of the Expert Panel and Sample Size

The number of experts in the Delphi technique were calculated by Thomas T. Macmillan found that more than 17 experts or equal resulted in a minimal error value (error level 0.02) [18]. Thus, the sample size was chosen for this study to be 17–21 participants. Purposive sampling was used for the selection of the TTM experts. The experts in the Delphi technique were selected from the list of expert committees of the Thai Traditional Medical Council with clinical experience in treatment for menstrual-cycle-related symptoms or menstrual disorders with TTM theoretical knowledge greater than 10 years.

#### 2.2.2. The Delphi Questionnaire

The first round of the Delphi Technique received an open-ended questionnaire, which was modified from a review of literature in TTM theory and interviewing TTM experts. This open-ended questionnaire was also used for interviewing 19 experts. After that, this questionnaire was modified into a structural questionnaire and was used in the 2nd round of Delphi questions. This 2nd round questionnaire would be sent back to the 19 experts to determine the importance level of items by a 5-point Likert rating scale; 1-strongly unimportant; 2- unimportant; 3-neutral; 4- important, and 5-strongly important. However, this structural questionnaire in the 2nd round was confirmed by the same 19 experts in the 3rd round. The time duration between 2nd and 3rd rounds was 2 weeks.

#### 2.2.3. The Delphi Analysis

In the 1st round, the open-ended questionnaire, a list of all contents was analyzed using content analysis. In the 2nd round, the importance level of each item of the draft questionnaire was evaluated and showed an outcome as median and interquartile range (IQR) [The IQR is the value of data distribution]. In the 3rd round (final round), the experts received a questionnaire which showed the median score and IQR of each item from the 2nd round. The experts were given an opportunity to revise their rating or retain the previous scores for confirmation of answers. The new median score and IQR of items in 3nd round were calculated to assess the level of consensus per question [19]. The items with the median score greater than or equal to 3.5 (high level of importance) and IQR less than or equal to 1.5 would be accepted to be important items that reached the consensus threshold [20]. These accepted items were used to establish the first draft MCSQ.

### 2.3. Evaluation of Validity and Reliability of the MCSQ

#### 2.3.1. Content Validation

The content validity index (CVI) of each item was evaluated by 6 experts who had experience in Thai traditional medicine for more than 10 years and were licensed to practice Thai traditional medicine. The expert panel provided a score of each item based on its relevance using a 4-point ordinal scale, with score 1 meaning “not relevant,” score 2 “somewhat relevant,” score 3 “quite relevant,” and score 4 “highly relevant.” At the end of each item was a clinical notation for the expert's comments. Relevance evaluation of each item under the pattern of TTM principles for menstrual-cycle-related signs and symptoms were calculated with the item-content validity index (I-CVI) and the scale-content validity index (S-CVI). The I-CVI of each item equal or greater than 0.83 was selected.

Subsequently, the revised MCSQ was used in a preliminary trial of 30 reproductive women (age 18–45 years with a clinically normal menstrual cycle) for evaluation on content, language, and time to complete the survey. Then, the questionnaires were revised and edited for clarity. Internal consistency was measured by Cronbach's alpha.

Through the results of the revised questionnaire, it was concluded that 63 items from 75 items were suitable. This questionnaire was divided into 2 sections: Section 1: Menstrual-cycle-related signs and symptoms (MCSs section) had 27 items; this section represented the signs and symptoms related to the menstrual cycle and the importance of identification of the internal organs and system that are the likely cause of the MCSs. Section 2: Associated factors (AF section); this section emphasizes personal information, behaviors, and other factors that affect the occurrence of MCSs. The AF section was divided into two parts. Part 1: Multiple-choice questions (14 items) that inquired about personal data, medical history, and gynecologic history, and Part 2: Rating scale questions (22 items) that queried the frequency of associated factors which affect the occurrence of menstrual-cycle-related signs and symptoms.

All items were rated on a 5-point rating scale as follows. For the question items related characteristics of menstrual blood, leucorrhea, and associated factors, the scale ranged from 0 “never” to 4 “always” to assess the frequency of the event. The scale of the question items indicated menstrual-cycle-related symptoms ranged from 0 “none” to 4 “extremely severe” to assess the severity of signs and symptoms.

#### 2.3.2. The Field-Testing of the Revised Questionnaire for Evaluation Validity and Reliability

Because the purported afflicted population is large and the true proportion is not known, the sample size of this study was calculated by Cochran's sample size formula. Assuming the maximum variability, which is equal to 50% (*p* = 0.5) and taking 95% confidence level with ±5% precision, the calculation for required sample size was set as follows: *p* = 0.5 and hence *q* = 1–0.5 = 0.5; *e* = 0.05; *z* = 1.96:

(1) $$\begin{array}{c} n_{0}=\frac{z^{2\;}pq}{e^{2}}\\ =\frac{{\left(1.96\right)}^{2\;}\left(0.5\right)\left(0.5\right)}{{\left(0.05\right)}^{2}}\\ =384.16\\ =385, \end{array}$$

where *n*_0_ is the sample size, *z* is found in statistical tables that contain the area under the normal curve, e.g., *z* = 1.96 for 95% level of confidence, *e* is the desired level of precision, *p* is the estimated proportion of an attribute that is present in the population, and *q* is a margin of error or it was calculated from 1 − *p*.

The sample size of the field testing was expanded by 10% of the power calculated sample size in anticipation of incomplete surveys with deficient answers or missing questionnaires. The questionnaires were equally distributed to 6 study sites (the sample size per study site: 428/6 = 71.3 = 72); therefore, the sample size of this study was 432. Comrey and Lee (1992) have described sample sizes of 300 as “good” and 500 as “very good” for factor analysis [21]. According to this guideline, the sample of 432 participants was deemed acceptable for exploratory factor analysis.

This study utilized purposive sampling from 6 regions with one province representing each region, namely, Pathum Thani (Central region), Prachin Buri (Eastern region), Phetchaburi (Western region), Loei (Northeastern region), Phayao (Northern region), and Songkhla (Southern region).

The revised MCSQs were distributed to 432 Thai women of reproductive age, 18–45 years, who were receiving services at the Thai Traditional Medicine department in hospitals or Thai traditional medical clinics. The inclusion criteria were: (1) female aged 18–45 years, (2) women having a menstrual cycle between 21 and 35 days, and (3) women that understand and communicate in the Thai language.

Exclusion criteria were: (1) amenorrhea (missing more than 2 menstrual cycles before participating in the study), (2) pregnancy or breast feeding within one year before participating in the study, (3) abdominal surgery within one year before participating in the study, (4) childbirth within one year before participating in the study, (5) history of abortion within one year before participating in the study, and (6) taking medication to treat a mental health disorder. The survey was conducted from December 2019 to February 2020.

### 2.4. Statistical Analysis

The data analysis was divided into two phases concomitant with the process of questionnaire development. The qualitative research in the first phase used content analysis. The second phase as quantitative research used the descriptive statistics including frequency or percentage, range, mean, and standard deviation (SD). The MCSQ (final version) score in each section and component were summarized as a percentage, mean, and SD.

The content validity of the questionnaire in quantitative research was assessed using the item-level content validity index (I-CVI) and the scale-level content validity index (S-CVI). The I-CVI is calculated as the number of experts providing a rating of 3 or 4 for each item divided by the total number of experts. The acceptable standard for the I-CVI should be more than 0.78 (considered “excellent”) and the S-CVI should be more than or equal to 0.9 [22].

Construct validity was evaluated using exploratory factor analysis (EFA). In this study, the MCSQ was split into two sections, and each section was evaluated for its construct validity separately. EFA was used to evaluate the validity of the component of the items mainly due to the TTM principal component aspects. The underlying structure of the revised MCSQ items was evaluated by principal component exploratory factor analysis using varimax rotation with Kaiser normalization. The criterion applied to retain the component was an eigenvalue ≥1.0 for that component. The factor loading for each item to meet this condition was set at 0.40 or more [23, 24].

The internal consistency or the reliability of the rating scale of the items and each component of the final MCSQ were measured by calculating Cronbach's alpha coefficient and corrected item-total correlation. The acceptable values of Cronbach's alpha were above 0.70 [25, 26] and the corrected item-total correlation was greater than or equal 0.20 [27].

### 2.5. Ethical Considerations

The expert panel and participants were approached by the researchers and invited to complete the questionnaire voluntarily through informed consent. They were informed about the objectives of the study, and that their personal information would be protected and would not be revealed to others. This study received approval from the Human Research Ethics Committee of Thammasat University No.1 (Faculty of Medicine) (MTU-EC-ES-1-162/61).

## 3. Results

### 3.1. Phase 1: Developing the Menstrual-Cycle-Related Symptoms Questionnaire (MCSQ)

#### 3.1.1. The Menstrual-Cycle-Related Signs and Symptoms Using TTM Principles

According to the official Thai traditional medicine textbook named “Paet-SaatSong-Kror” (in Thai language), the human body consists of four elements: Earth element (all organs), water element (all liquid in the body such as blood, saliva, urine, etc.), wind element (the circulation of blood, the signal transmission of the nervous system, the movements of the body, and the transportation of gases in the respiratory system), and the fire element (such as the metabolic system, endocrine system, chemical mediators). All functions of the organ and liquid system are controlled by the wind and fire elements [28]. Vedcha-SukSaa textbook (in Thai language) states that disorders or diseases are caused by the impaired function of elements. The causes of disease are age, environment (hot and cool weather), behaviors (eating, sleeping, excreting, bodily movements), tastes and types of food and drinks, and emotion. All of these factors affect the balance elements in the body [29].

Thai traditional medical scripture regarding women's diseases and menstruation called “Mahaa-Chotarat (in Thai language)” states that the irregularity of blood and menstruation are the principal causes of illnesses or diseases in women. The fire and wind elements are the basis regulators for all functions of organs (physical and mental processes) including the smooth flow of blood throughout the body. Blood is the water element that circulates and nourishes various organs within the body. Accordingly, if the function of the fire and wind elements is impaired, the blood circulation will be interrupted affecting various emotional and physical symptoms. Menstruation is directly related to the blood or water element, the wind element, and the fire element. Before menstruation, the body has more power of the fire, wind, and water (blood) elements than usual. The increasing power of these elements affects the function of organs inside the body. According to TTM principles, MCSs are the group of physical and/or emotional symptoms that occur before and/or during the menstrual period and disappear before or after the onset of menstruation. Most MCSs are caused by the following organ malfunctions: muscle and liver, joint and bone, gallbladder (in the TTM principles, it is also responsible for controlling heat), skin, heart, and intestine and mesentery (in TTM principles organs work together). Moreover, there will be irregular characteristics of menstrual blood and leucorrhea (color, texture, and smell) that indicate that the blood and uterus have a disorder. These MCSs are caused by an impaired function of the fire and wind elements affecting the malfunctions of blood and related organs. Accordingly, TTM clinicians use these signs and symptoms to examine and find the organ or system inside the body, which is the origin of the disorder.

According to TTM principles and the TTM clinician tenet, the MCSs are influenced by several external and internal factors, as shown in Table 1.

**Figure 2:**
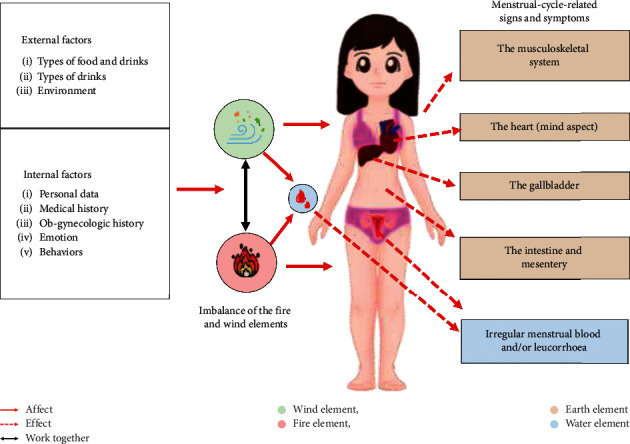
TTM theoretical concept of menstrual-cycle-related signs and symptoms.

These factors exert an effect on the function of the fire and wind elements, and blood, leading to an increase in the fire element and/or the wind element causing the poor circulation of blood and giving rise to menstrual-cycle-related symptoms and signs. The mechanism of MCSs according to the principle of TTM is shown in Figure 2.

Consequently, this study utilized the described theoretical framework to guide the development of the questionnaire for assessing the MCSs. The two important issues of this questionnaire were the MCSs and associated factors that can reflect the origin of the disorder.

#### 3.1.2. Questionnaire Development via Delphi Method

Nineteen out of the 21 experts agreed to participate in the Delphi study and all of them completed the three rounds of the Delphi questionnaire; the flow of the participation is shown in Figure 1. The expert panel was selected from different TTM operational levels comprising 12 TTM clinicians from clinics or hospitals and 7 experts from the university. The characteristics of TTM experts are given in Table 2.

The expert panel was asked about the appropriate domains and items associated with assessing MCSs based on the TTM principle. There was a consensus on the important items including physical and/or emotional symptoms that occur before or during menstruation, the characteristics of menstrual blood and leucorrhea, and the factors affecting MCSs were determined to be the appropriate clinical essentials and items for this assessment.

The final round contained a total of 75 items, each item having a median ≥3.50 (high level of importance) and IQR ≤ 1.50. The summarized detail of the questionnaire is shown in Table 3. In section 1: MCSs section, because characteristics of menstrual blood and leucorrhoea have similar content in some items, experts recommended including items with consistent content to be one item to reduce the number of items and redundancy, resulting in 9 remaining items of characteristics of menstrual blood and leucorrhoea. The expert panel suggested that these items should use the frequency score to assess the severity of menstrual blood and leucorrhoea (if a woman always has irregular menstrual blood or leucorrhea, indicating the blood was terrible). In Section 2: Associated factors section, due to the associated factors being so detailed and involving a lengthy number of items, experts recommended including items with consistent content to be one item to reduce the number of items and redundancy, resulting in the 35 remaining items of associated factors. Thus, the first draft MCSQ included 61 items in two sections: the menstrual-cycle-related signs and symptoms section (the MCSs section) had 26 items and the associated factors section (the AF section) had 35 items. Each section incorporated a number of subcategories according to the theoretical framework (the content above) and the expert consensus.

The MCSs section subcategories focus on the important signs and symptoms that reflect the problematic internal system or organ, including the following five dimensions: (1) the signs reflect the problem of the blood in the uterus (characteristics of menstrual blood and leucorrhea), (2) the symptoms reflect the problem of the musculoskeletal system, (3) the symptoms reflect the problem of the intestine, (4) the symptoms reflect the problem of the gallbladder (it is the controller of the heating system inside the body), and (5) the symptoms reflect the problem of heart (mind aspect).

The AF section subcategories addressed aspects of the causes that affect the occurrence of MCSs, including the following seven dimensions: (1) personal data, (2) medical history, (3) Ob-Gynecologic history, (4) the types of food and drink, (5) The behaviors and health problems, (6) the environment (hot and cool), and (7) the emotion and feeling. The details of items of MCSQ in the 3rd round of the Delphi method are shown in Supplementary Table 1.

### 3.2. Evaluation of Validity and Reliability of the MCSQ

#### 3.2.1. Content Validity of the MCSQ

After finishing the Delphi method, the content validity was performed by five experts to evaluate the content validity of the first draft MCSQ. All items (61 items) of this questionnaire showed excellent content validity (I-CVI = 0.83 and 1.00). The average scale content validity (S-CVI/AVE) was 0.98, above the cut-off of 0.90. There were 2 additional items as recommended by experts. The items were later adjusted or changed for clarity according to experts' suggestions. Thus, the revised MCSQ included 63 items in two sections. The items with CVI of the revised MCSQ are shown in Supplementary Table 2. The pretesting of the revised MCSQ showed Cronbach's *α* coefficient was 0.85 overall, indicating good internal consistency and appropriate item homogeneity.

#### 3.2.2. The Evaluation of MCSQ by Construct Validity

The questionnaires were distributed to 432 reproductive-age Thai women. Two women were excluded and one woman did not complete the questionnaire; therefore, there were 429 participants or useful questionnaires (response rate of 99.31%). The mean age of the participants was 26.00 ± 7.27 years (aged 18–45 years). The majority of participants were single (81.4%), 15.8% of the participants were married, and the remaining 2.9% was either separated or widowed. The participants reported menarche at 11.00 ± 4.20 years old. The general information of participants is shown in Table 4.

Eight items of the revised draft MCSQ were removed before analyses via EFA because 2 items of menstrual-cycle-related symptoms had high skewness and kurtosis values (2 and 4, respectively), showing nonnormal distribution of the data, and six items of associated factors had low internal consistency (the items were negative or less than 0.2). The revised MCSQ consisted of 55 items in two sections: the MCSs section had 25 items and the AF section had 30 items. In the AF section, only 16 items of the AF in the rating scale part were evaluated using EFA.

This study performed principal component analysis (PCA) to explore the factor structure of the MCSQ. The factor analysis was conducted in each section of the questionnaire. *The MCSs section* (section 1), the value of the Kaiser–Meyer–Olkin (KMO) was 0.89 and Bartlett's test of sphericity (*χ*^2^ = 3342.770, df = 300, *p* value ≤ 0.001) indicated that the data had adequacy and was appropriate for the analysis model. The AF section (section 2), the data also met the Kaiser–Meyer–Olkin's sample adequacy criteria (0.75, minimum acceptable level 0.60), as well as those for Bartlett's test of sphericity (*χ*^2^ = 1154.917, df = 120, *p* value ≤ 0.001) for the appropriateness of using the analysis model. Thus, it was acceptable to adopt a factor analysis to test the construct validity of this questionnaire.

The results of EFA, with PCA as the extraction method, and Varimax, with Kaiser normalization as the rotation method, revealed that the MCSs section consists of 5 components that can explain 51.27% of the total variance, and the AF section consisted of 5 components that can explain 54.21 % of the total variance. Mean and SD of each item, each component; factor loading; commonalities; eigenvalues; and variances of each section are presented in Tables 5 and 6. Section 1: MCSs section, as shown in Table 5, Component 1 was “the problem of blood in the uterus,” Component 2 was “the musculoskeletal system,” Component 3 was “the heart (mind aspect),” Component 4 was “the digestive system (intestine and mesentery),” and Component 5 was “the irregular menstrual blood.”

Section 2: AF section, as shown in Table 6, Component 1 was “the emotion and feeling,” Component 2 was “the type of drinks,” Component 3 was “the type of food,” Component 4 was “the environment,” and Component 5 was “the behavior and health problem.” The intercorrelation of subscale of both sections is shown in Supplementary Tables 3 and 4.

There were five items that belong to the inappropriate component according to the TTM theoretical conceptual construct and they were removed before calculating Cronbach's *α* coefficient of each component. Cronbach's *α* coefficient for each section was 0.87 (the MCSs section) and 0.69 (the AF section). Cronbach's *α* coefficient of subscale of both sections are given in Tables 5 and 6.

Finally, the MCSQ consisted of 49 items divided into two sections, 35 items in ten components which were rating-scale questions (MCSs section 23 items in five components and AF section 12 items in five components), and 14 multiple-choice questions (AF section). The complete list of items and their components in the final MCSQ are given in Table 7.

#### 3.2.3. The MCSQ Score of Participants

Accordingly, the MCSQ is the screening tool to assess the severity of MCSs, which indicates the problematic organ or system inside the body, and assesses the causes of the disorder. The total score of the MCSs section was the sum of the scores for the 23 items (score range 0–92). The 25th percentile was defined as the lowest score group. The 75th percentile was defined as the highest score group. The mean score of MCSs section was 25.78 ± 12.01, indicating that the participants had the severity of menstrual-cycle-related signs and symptoms at a mild level according to this study's criteria. This study showed that all participants had at least one organ or system which had a problem related to the menstrual cycle. About 99.07% of the participants had menstrual-cycle-related symptoms caused by the musculoskeletal system. The total score of the AF section was the sum of the scores for the 12 items (score range 0–48). The mean score of associated factors section was 21.40 ± 6.24 (the score at a fair level according to this study's criteria). The MCSQ scores of each section and subscale are given in Table 8, and the score interpretation is given in Supplementary Table 5.

In addition, five components of the AF section were tested by the extreme groups of MCSs section using the unpaired Student's *t*-test. The AF score comparisons between the lowest group and highest group are given in Table 9. The total score in the component “Emotion and feeling,” “Types of food,” “Environment,” “Behavior and health problem,” and the total score of AF section had statistically significant differences between the lowest and highest score groups. This study revealed that the total score of the MCSs section has a moderate correlation with the total score of the AF section (*r* = 0.41, *p* value ≤ 0.001).

## 4. Discussion

The purpose of this study was to develop and evaluate the initial validity and reliability of the menstrual-cycle-related symptoms questionnaire (MCSQ) based on TTM principles. The MCSQ is a questionnaire for assessing the severity of MCSs and associated factors in reproductive-age women. Moreover, this tool can indicate the problematic organ or system inside the body. Because TTM knowledge regarding assessing the menstrual-cycle-related signs and symptoms is unclear, this study attempted to develop a questionnaire using mixed-method research. This is the first study to develop a MCSQ based on TTM principles for reproductive-age Thai women. The results are supportive of initial evidence of its reliability and validation in the healthy reproductive-age Thai women. This study used items from the interviews and the Delphi method in which the participants were TTM clinicians and experts; information was obtained directly from experienced TTM clinicians and experts, providing a perspective of the clinical experience.

### 4.1. Thai Traditional-Medicines-Related MCSs

According to TTM principles and tenets, MCSs are the results of dysfunctions or imbalance of the fire and wind elements, and the impairment of blood that affects the function of organs inside the body [10]. The results of the Delphi study revealed that almost all MCSs are mentioned in TTM scriptures, and the expert panel discussed that these MCSs were sufficient as these were common symptoms that should be treated (breast tenderness and abdominal cramps were added because they are common symptoms in the present day). “Fever before or during menstruation” is an important menstrual-cycle-related symptom based on TTM theory; it is associated with the severity of menstrual problems. Moreover, this symptom indicates the dysfunction of the fire element, which can be compared with changes in hormonal levels of estrogen and progesterone that cause imbalances in the body and cause the body to have less immunity to diseases [30]. In principles of both Buddhism and TTM, the heart is a living embodiment of mind, and mind is one of nature that knows emotion. This is the reason why the symptoms of the heart system described in TTM scripture are further related to emotion and psyche than the symptoms of heart disease [31]. According to the knowledge of TTM clinicians, all of the menstrual-cycle-related symptoms often are related to the behaviors or activity of each person—if a woman sits for a long time during a day or works hard, it causes muscle strain and leads to the occurrence of musculoskeletal symptoms during the menstrual period, or if a woman often has a negative emotion (depression, stress, irritability), it shows that the elements in the heart have impairment, which leads to the occurrence of negative emotions during the menstrual period. Furthermore, the associated factors were the indicators necessary for TTM pattern assessment on menstrual problems. Associated factors consist of the types of food and drink, taste and flavor of food and drink, behavior, health problems, emotion, and environment. All of them can influence menstruation, e.g., strong-flavored foods, ice-beverages, hard work, hotness, coldness, bad emotion, and high stress. These were generally referred to as causes of diseases according to TTM principles [32]. These associated factors will affect the menstrual cycle if they occur regularly.

### 4.2. MCSQ Development, Validity, and Reliability

This study performed EFA to explore the latent structure of the MCSQ. The factor analysis was conducted separately in each questionnaire's section because each section assesses a different dimension—the signs and symptoms, and the causes of the disorder. Two items of MCSs that were removed because they had high skewness and kurtosis values were “Rash/urticaria/bruise or a burning feeling on the skin” and “Nausea and/or vomiting.” The researcher hypothesized that both of these items were not the common symptoms in this study's participants. Construct validity was used to evaluate whether or not the questionnaire corresponded to the theoretical design upon which the questionnaire was based [23]. Results of the analysis showed that the structure of this tool was rather in accordance with the theoretical framework (Figure 2). Some symptoms may appear in different components, hot and cold flushes/fever, fatigue, and waking up with fright/insomnia as examples, which are the symptoms that reflect a problem of the gallbladder according to the TTM scripture, but it can occur in the musculoskeletal system and the heart too. Accordingly, it might be the reason why “the problem of the gallbladder” component did not happen [10, 33]. Nevertheless, some components and item of each section in the MCSQ were not satisfactory including the following:

1. The MCSs section: the questions which asked about the “problem of the blood in the uterus” and “irregular menstrual blood” were combined into one answer because each question involved overlap in the irregular characteristics of menstrual blood and leucorrhea.
2. The AF section: the “types of food” component and the “types of drink” component should be combined into one component because both components have similar underlying concepts, i.e., the taste of food and drink affects the balance of the fire and wind elements, and blood.
3. From the PCA result, the “headache or dizziness” item and “pubic symphysis pain or burning feeling in the vagina” item should belong to the musculoskeletal system component rather than “irregular menstrual blood” component, or “characteristics of menstrual blood and leucorrhea” component. And, four items of the associated factors, including “eating fried food, oily food, bakery products” item, “drinking alcoholic drinks” item, “sitting for a long time during a day” item, and “travel to cities or places with a different climate from your current place of residence” item, belong to the wrong component. Considering this contradiction, these items were removed from the questionnaire. However, the removed items in this study should be further modified and could be used for further reflective development in the next study.

The internal consistency was analyzed for reliability within each component. Cronbach's *α* coefficient of two components in the MCSs section (“the digestive system” component and “irregular menstrual blood” component), and four components in the AF section (except emotion and feeling component) was relatively low (0.32 to 0.62). This would suggest that the items of each component did not correlate very well overall. The lack of correlation may also be due to each question involving observable symptoms or characteristics, difficulty in answering the question with clarity, or insufficient number of items for each component to determine a clear relationship.

The MCSQ score norm could not be used because our population was not representative of the Thai women in general. Results of the MCSQ score were just a preliminary analysis for determining the score of these participants. This questionnaire needs more complete validation to determine a normal standardized score of the questionnaire. The AF score comparisons between the lowest group and the highest group were evaluated differently between groups. The result showed significant statistical differences in four components, and the lowest group and the highest group had total AF scores with significant statistical differences. The “type of drinks” was, however, not statistically different between groups. The researcher hypothesized that most participants have a mild to moderate level of severity, resulting in the score of this component not being different between groups and the score of some components being only slightly different. Thus, further study of this hypothesis needs to be tested and proven in women with and without the MCSs.

## 5. Limitations

This study has some limitations as the Delphi technique is a new method for TTM research. The Delphi method is a questionnaire development technique based on purposive sampling; it has the potential risk for bias in expert selection and representativeness [34]. However, the study was designed to set distinct criteria for expert selection to ensure some degree of homogeneity among the experts.

Due to practical limitations, content validity was tested only on a small group of experts, that is, using the recommended lowest number of experts (6 persons) [22], and only one round of expert reviews was performed.

The limitations of the MCSQ in the reliability and validity paragraph test include: (1) using a retrospective self-reported measure where there is a risk of incorrect recall and inflated or deflated answers to reflect the severity of symptoms and frequency of behavior that were associated factors. Participants could possibly forget the exact severity level of the symptom or frequency of the behavior, when required to recall after a long period (six months). (2) The MCSQ consisted of many items and participants had to spend a long time to complete it (about 20 minutes), which could make them bored and potentially not paying close enough attention to their responses. (3) Due to the fact that the MCSQ was designed for reproductive-age women who want to assess their menstruation, the survey was initially conducted with healthy participants who had normal menstruation. Hence, the samples in this study may not be a good representation of the overall population of Thai women, and its specificity to a population who had menstrual problems is unknown and must be tested. Lastly, because this questionnaire is multidimensional and designed according to the TTM theoretical framework, to complete validation, this questionnaire should be assessed for the confirmatory factor analysis (CFA) to test-retest reliability. Moreover, the norm and scoring system should be determined. Further studies should recruit various groups of Thai reproductive-age women, e.g., women who have menstrual problems, to ensure its generalization, and clinical research should be applied and evaluated.

## 6. Conclusions

The MCSQ is the first menstrual questionnaire that was developed using the TTM principles as the framework. This questionnaire was developed for reproductive-age women. The MCSQ is a self-assessment questionnaire consisting of 49 items divided into two sections, MCSs and associated factors. The questionnaire included 35 items in ten components which were rating-scale questions and 14 multiple-choice questions. The MCSQ shows moderate to high content validity of individual items (I-CVI ranges: 0.83–1.00) and high content validity of the overall questionnaire (S-CVI/AVE = 0.98). The construct validity testing showed the structure of this tool rather was in accordance with the principle of TTM. The internal consistency of each component was moderate to good (alpha value range: 0.32 and 0.82) and this could be further optimized. As a result, this tool needs more extensive validation and rigorous verification before it is used in future research and clinical practices. However, the developed MCSQ will be a useful guideline for clinical treatment and encourages women's personalized self-care for either the reduction of the severity of the MCSs to having normal menstruation and/or the increasing of the effectiveness of personalized therapeutic treatment.

## Figures and Tables

**Table 1:** The external and internal factors affecting MCSs according to TTM knowledge.

Factors affecting MCSs according to TTM knowledge [10, 29]	Example of the factors
*Internal factors*	
Personal data	Age, major elements in the human body, obesity

Medical history	Underlying illness or health problems (migraine, asthma, atopic dermatitis, insomnia, constipation, etc.), accident, abdominal surgery
Ob-gynecologic history	Pregnancy and childbirth history, postpartum care, abortion, curettage, contraceptive use
	Gynecological disease, age at menarche, first time menstrual-cycle-related symptoms began, family history (grandmother/mother/sister/twin)
Emotion	Angry, irritability, anxiety, stress, sad, depressed
Behaviors	Skipping meals, being in the same position for a long time, working excessively, sleep deprivation, often sleeps late

*External factors*	
Types of food	Uncooked food or raw food, strong-flavored food, preserved food, fried food, oily food
Types of drink	Energy drinks, carbonated drinks, iced drinks, frappe, coconut juice, alcoholic drinks, caffeinated drinks
Environment	Contact with chemicals, hot weather, cold weather, climate change immediately

**Table 2:** Characteristic of the TTM expert (*N* = 19).

Characteristics	*N* (%)
*Gender*	
Male	7 (37%)
Female	12 (63%)

*Clinical experience*	
5–10 years	11 (58%)
11–20 years	5 (26%)
>20 years	3 (16%)

*Current position*	
TTM clinician	
Operational level	6 (32%)
Experienced level	3 (16%)
Senior level	3 (16%)
Head of Department of Thai Traditional Medicine in university	2 (11%)
University professor	5 (26%)

**Table 3:** Summary of the third-round questionnaire (Delphi method).

Section 1: Menstrual-cycle-related signs and symptoms (total 32 items)	
Subsection 1.1 Characteristics of menstrual blood and leucorrhoea (15 items)	The signs reflect the problem of the blood in the uterus
3 items: 4.00* *>* *median* *≤* *4.50, IQR* *≤* *1.50	The irregular menstrual blood flow (3 items)
12 items: median* *>* *4.50, IQR* *≤* *1.50	The irregular color of menstrual blood (3 items)
	The irregular texture of menstrual blood (2 items)
	The irregular smell of menstrual blood (2 items)
	Menstrual blood clots (2 items)
	The irregular characteristics of leucorrhea (3 items)
Subsection 1.2 Menstrual cycle-related symptoms (17 items)	The symptoms reflect the problem of the musculoskeletal system (7 items)
7 items: 3.50* *>* *median* *≤* *4.00, IQR* *≤* *1.50	The symptoms reflect the problem of the intestine and mesentery (4 items)
9 items: 4.00* *>* *median* *≤* *4.50, IQR* *≤* *1.50	The symptoms reflect the problem of the gallbladder (3 items)
1 item: median* *>* *4.50, IQR* *≤* *1.50	The symptoms reflect the problem of heart (mind aspect) (3 items)

*Section 2. Associated factors (total 43 items)*	
Internal factors (28 items)	Personal data (3 items)
4 items: 3.50* *>* *median* *≤* *4.00, IQR* *≤* *1.50	Medical history (3 items)
15 items: 4.00* *>* *median* *≤* *4.50, IQR* *≤* *1.50	Ob-gynecologic history (8 items)
9 item: median* *>* *4.50, IQR* *≤* *1.50	The behaviors and health problems (11 items)
	The emotion and feeling (3 items)
External factors (15 items)	
4 items: 3.50* *>* *median* *≤* *4.00, IQR* *≤* *1.50	The types of food and drink (11 items)
11 items: 4.00* *>* *median* *≤* *4.50, IQR* *≤* *1.50	The environment (hot and cool) (4 items)

**Table 4:** General information of participants (*n *=* *429).

Characteristic	*n* (%)
Mean age ± SD (years)	26.00 ± 7.27
Mean age of menarche (SD) (years)	11.00 ± 4.20
Marital status	
Single	349 (81.4%)
Married	68 (15.8%)
Widowed/divorced/separated	12 (2.9%)

Education	
Lower than bachelor degree	53 (12.4%)
Bachelor degree	345 (80.4%)
Graduate degree	31 (7.2%)

Menstrual disorder in family	
Yes	57 (13.3%)
No	252 (58.7%)
Unknown	120 (28.0%)

Contraceptive use	
Never used	322 (75.0%)
Used to, but no longer using	75 (17.5%)
Used to and still in use today	32 (7.5%)

**Table 5:** Mean, SD, factor loading communalities, eigenvalues, variances, and Cronbach's *α* coefficient for the menstrual-cycle-related signs and symptoms section.

Menstrual-cycle-related signs and symptoms	Score		Communalities					Components
	Mean	SD	1	2	3	4	5	
*The problem of blood in the uterus*								
1. Vaginal discharge is an abnormal color with a bad smell	0.52	0.93	0.709					0.534
2. A lot of vaginal discharge and vaginal discharge is eggy, clear, stretchy, or thick white	1.66	1.25	0.660					0.483
3. Before the menstrual period, have vaginal discharge and vaginal itching	0.71	0.96	0.654					0.559
4. Menstrual blood smells stronger than usual or smells like rotten meat	0.55	0.82	0.643					0.528
5. Thick texture menstrual blood	0.99	1.10	0.616					0.480
6. Menstrual blood looks like egg whites	0.90	1.05	0.537					0.375
7. A lot of menstrual blood clots are released every day or are of large size (greater than or equal to 2 centimeters).	0.93	1.01	0.471					0.369
8. Pubic symphysis pain or burning feel in the vagina*∗*	0.51	0.81	0.419					0.359

*The musculoskeletal system*								
9. Breast pain	1.68	0.99		0.722				0.557
10. Abdominal cramps	1.85	1.13		0.704				0.622
11. Muscle pain/lower back pain	1.89	1.00		0.695				0.603
12. Hot and cold flushes or fever	0.87	0.88		0.562				0.508
13. Fatigue	1.42	1.06		0.551				0.573
14. Joint pain/bone pain	0.94	1.10		0.419				0.446

*The heart (mind aspect)*								
15. Anxiety and/or tension	1.05	1.05			0.840			0.783
16. Depression and/or crying	1.28	1.13			0.808			0.769
17. Irritability and/or anger	1.77	1.02			0.591			0.620
18. Insomnia or waking up with a fright	0.77	0.99			0.499			0.438

*The digestive system (intestine and mesentery)*								
19. Colic in the abdomen or flanks	0.44	0.72				0.669		0.537
20. Loose/watery stools five or six times a day	0.65	0.87				0.626		0.449
21. Abdominal bloating	0.98	1.04				0.601		0.504

*Irregular menstrual blood (the impairment of blood)*								
22. Light or heavy menstrual bleeding throughout the cycle (using less than 2 pads per day or more than 4 pads per day).	1.26	1.05					0.612	0.458
23. Many colors of menstrual blood in one period	1.72	1.34					0.605	0.456
24. Headache/dizziness*∗*	1.10	1.06					0.532	0.532
25. Menstrual blood color is pale red, bright red, orange, dark red, dark brown, or black.	0.98	1.02					0.429	0.273

*Eigenvalue*			3.18	3.05	2.73	1.99	1.83	
% of variance explained			12.71	12.20	10.93	7.98	7.45	
% of variance explained overall					51.27			

*Reliability*								
Cronbach's *α* coefficient			0.77	0.80	0.82	0.58	0.45	

*∗*It was the item that belongs to the wrong component according to the theoretical concept and was removed before calculating Cronbach's *α* coefficient of each component.

**Table 6:** Mean, SD, factor loading communalities, eigenvalues, variances, and Cronbach's *α* coefficient for the associated factors section.

Associated factors	Score		Components					Communalities
	Mean	SD	1	2	3	4	5	
*Emotion and feeling*								
1. You are irritable or angry	1.72	1.01	0.848					0.749
2. You are bored, depressed, or in despair	1.42	1.06	0.789					0.662
3. You feel anxious or worried	1.98	1.11	0.789					0.722
*Types of drinks*								
4. You like to drink ice-beverages or frappe	2.77	1.05		0.778				0.632
5. You like to drink caffeine beverages (e.g., chocolate, tea, carbonated beverage, energy drinks, coffee)	2.09	1.30		0.763				0.634
6. You like to eat fried food, oily food, bakery products, e.g., streaky pork, bacon, cheese, bread, cakes*∗*	2.25	0.93		0.661				0.515

*Types of food*								
7. You eat preserved food and/or uncooked food, e.g., fruit preserves, sashimi, and medium to raw meat	1.49	0.93			0.742			0.572
8. You like to eat strong-flavored foods, e.g., extremely spicy, extremely sour	2.32	1.12			0.679			0.632
9. You like to drink alcoholic drinks*∗*	0.78	0.93			0.578			0.394

*Environment (hot and cold)*								
10. You sit for a long time during the day (more than 30 minutes/time)*∗*	2.14	1.17				0.676		0.518
11. In one day, you must enter and exit the area with temperature differences (hot and cold)	2.17	1.21				0.563		0.383
12. You work or stay in a bad environment for a long time a day (in the area too hot or cold/exposed to or inhaling chemicals)	1.20	1.14				0.509		0.372

*Behavior and health problem*								
13. You have sleep problems, e.g., insomnia, waking up with a fright in the middle of the night	1.54	1.06					0.600	0.603
14. You travel to cities or places with a different climate from your current place of residence*∗*	0.76	0.80					0.522	0.468
15. You work hard (using a lot of energy or muscle power)	1.27	0.95					0.494	0.451
16. You have constipation	1.45	1.10					0.425	0.364

*Eigenvalue*			2.39	1.84	1.59	1.55	1.31	
% of variance explained			14.97	11.49	9.92	9.67	8.17	
% of variance explained overall					54.21			

*Reliability*								
Cronbach's *α* coefficient			0.81	0.62	0.53	0.38	0.32	

*∗*It was the item that belongs to the wrong component according to the theoretical concept and was removed before calculating Cronbach's *α* coefficient of each component.

**Table 7:** The complete list of items and their components in the final MCSQ.

Section 1. Menstrual-cycle-related signs and symptoms (rating scale)
1.1 The problem of blood in the uterus
1. You have thick texture menstrual blood.
2. Your menstrual blood looks like egg whites.
3. Your menstrual blood smells stronger than usual or smells like rotten meat.
4. You have a lot of menstrual blood clots which are released every day, or large-size clots (greater than or equal to 2 centimeters).
5. You have a lot of vaginal discharge that you need to put on a sanitary pad and your vaginal discharge is eggy, clear, stretchy, or thick white.
6. Your vaginal discharge is an abnormal color with a bad smell (abnormal color: yellow, green, brown or blood).
7. Before the menstrual period, you have vaginal discharge and vaginal itching.

1.2 Irregular menstrual blood
8. You have light or heavy menstrual bleeding throughout the cycle (using less than 2 pads per day or more than 4 pads per day).
9. Your menstrual blood color is pale red, bright red, orange, dark red, dark brown, or black.
10. In one period, you have many colors of menstrual blood.

1.3. The musculoskeletal system
11. Hot and cold flushes or fever
12. Muscle pain/lower back pain
13. Joint pain/bone pain
14. Fatigue
15. Breast pain/tender breasts
16. Abdominal cramps

1.4. The digestive system (intestine and mesentery)
17. Loose/watery stools five or six times a day
18. Colic in the abdomen or flanks
19. Abdominal bloating/abdominal discomfort

1.5 The heart (mind aspect)
20. Waking up with a fright/insomnia
21. Irritability and/or anger
22. Depression and/or crying
23. Anxiety and/or tension

Section 2. Associated factors (rating scale)
2.1. Emotion and feeling
24. You feel anxious or worried.
25. You are irritable or angry.
26. You are bored, depressed, or in despair.

Section 2. Associated factors (rating scale)
2.2. Types of drink
27. You like to drink ice-beverages or frappe.
28. You like to drink caffeine beverages (e.g., chocolate, tea, carbonated beverage, energy drinks, coffee).

2.3. Types of food
29. You like to eat strong-flavored foods, e.g., extremely spicy, extremely sour.
30. You eat preserved food and/or uncooked food, e.g., fruit preserves, sashimi, and medium to raw meat.

2.4. Environment
31. You work or stay in a bad environment for a long time a day (in the area too hot or cold/exposed to or inhaling chemicals).
32. In one day, you must enter and exit the area with temperature differences (hot and cold).

2.5. Behaviors and health problems
33. You work hard (using a lot of energy or muscle power).
34. You have sleep problems, e.g., insomnia, waking up with a fright in the middle of the night.
35. You have constipation.

Section 2. Associated factors (multiple choice)
2.6. Personal data
36. How old are you?
37. Date and time of birth
38. Weight and height

2.7. Medical history
39. What is your health problem or underlying disease?
40. Have you ever had an accident that injured your lower back or lower abdomen?
41. Have you ever had abdominal surgery?

2.8. Ob-gynecologic history
42. How old were you when your first period start?
43. Have you ever given birth to a child?
44. What postpartum care did you have?
45. Have you ever miscarried?
46. Have you ever had a curettage?
47. Have you ever used hormonal birth control?
48. Has your grandmother or mother had a menstrual disorder history?
49. When did your menstrual symptoms first occur?

**Table 8:** The final MCSQ scores of each section and subscale of participants (*n* = 429).

Section	No. of items	Total score	Mean ± SD (min, max)	Percentage score	Level *∗* number (percentage)			
					None	Mild	Moderate	Severe
1. Menstrual-cycle-related signs and symptoms section	23	92	25.78 ± 12.01 (3.72)	28.02	0	294 (68.5)	134 (31.2)	1 (0.2)
1.1. The problem of blood in uterus	7	28	6.24 ± 4.62 (0.22)	22.29	31 (7.2)	309 (72.0)	85 (19.8)	4 (0.9)
1.2. Irregular menstrual blood	3	12	3.96 ± 2.37 (0.11)	33.00	36 (8.4)	218 (50.8)	164 (38.2)	11 (0.6)
1.3. The musculoskeletal system	6	24	8.65 ± 4.37 (0.23)	36.04	4 (0.9)	221 (51.5)	180 (42.0)	24 (5.6)
1.4. The digestive system (intestine and mesentery)	3	12	2.07 ± 1.97 (0.10)	17.00	116 (27.0)	262 (61.1)	46 (10.7)	5 (1.2)
1.5. The heart (mind aspect)	4	16	4.86 ± 3.37 (0.16)	30.38	35 (8.2)	230 (53.6)	145 (33.8)	19 (4.4)

					Very good	Good	Fair	Bad
2. Associated factors section	12	48	21.40 ± 6.24 (3.40)	44.58	0	97 (22.6)	317 (73.9)	15 (3.5)
2.1. Emotion and feeling	3	12	5.13 ± 2.71 (0.12)	42.75	18 (4.2)	168 (39.2)	194 (45.2)	49 (11.4)
2.2. Types of drinks	2	8	3.81 ± 1.69 (0.8)	47.63	9 (2.1)	92 (21.4)	257 (59.9)	71 (16.6)
2.3. Types of food	2	8	4.85 ± 2.01 (0.8)	60.63	3 (0.7)	56 (13.1)	199 (46.4)	171 (39.9)
2.4. Environment	2	8	3.37 ± 1.84 (0.8)	42.13	31 (7.2)	110 (25.6)	239 (55.7)	49 (11.4)
2.5. Behaviors and health problems	3	12	4.25 ± 2.03 (0.12)	35.42	10 (2.3)	240 (55.9)	170 (39.6)	9 (2.1)

*∗*According to the criteria for interpreting results of each section that was set by the researcher.

**Table 9:** The associated score comparison between the lowest and highest groups.

Subscale	Lowest group		Highest group		t	*p* value
	n	Mean (SD)	n	Mean (SD)		
Emotion and feeling	117	3.73 (2.36)	113	6.54 (2.70)	−8.43	<0.001
Types of food	117	3.47 (1.55)	113	3.92 (1.82)	−2.02	<0.05
Types of drink	117	4.48 (1.88)	113	5.00 (2.11)	−2.01	NS
Environment	117	3.02 (1.82)	113	3.68 (1.83)	−2.75	<0.05
Behavior and health problem	117	3.55 (2.02)	113	5.39 (1.97)	−7.00	<0.001
Total score of associated factors	117	18.24 (5.98)	113	24.54 (5.93)	−8.02	<0.001

SD = standard deviation, NS = not significant (*p* value ≥ 0.05).

## Data Availability

The data came from the questionnaire survey results which can be made available upon request.

## Abbreviations

AF: Associated factors
CFA: Confirmatory factor analysis
COPE: Calendar of premenstrual experiences
CVI: Content validity index
EFA: Exploratory factor analysis
I-CVI: Item-content validity index
MCSQ: Menstrual-cycle-related symptoms questionnaire
MCSs: Menstrual-cycle-related signs and symptoms
MDQ: Menstrual distress questionnaire
MSQ: Menstrual symptom questionnaire
PSST: Premenstrual symptoms screening tool
S-CVI: Scale-content validity index
S-CVI/AVE: Average scale content validity
TTM: Thai traditional medicine.

## Supplementary Materials

Supplementary Materials Table 1 shows median, interquartile range, and percentage of expert agree of each item in the third-round questionnaire (Delphi method). Table 2 shows the content validity index of the first draft MCSQ. Table 3 shows an intercorrelation of the subscale of the menstrual-cycle-related signs and symptoms section (Pearson's r). Table 4 shows an intercorrelation of the subscale of the associated factors section (Pearson's r). Table 5 shows the interpretation of total score and the score of each domain in the final MCSQ.
