# Supplementary material for: Development of an Assessment Tool of Menstrual-Cycle-Related Signs and Symptoms Based on Thai Traditional Medicine Principles for Evaluation of Women's Health
Source: Evid Based Complement Alternat Med. 2021 May 21;2021:9977773. doi: 10.1155/2021/9977773 (PMC8163529; doi:10.1155/2021/9977773)
Supplement: Supplementary Materials — Table 1 shows median, interquartile range, and percentage of expert agree of each item in the third-round questionnaire (Delphi method). Table 2 shows the content validity index of the first draft MCSQ. Table 3 shows an intercorrelation of the subscale of the menstrual-cycle-related signs and symptoms section (Pearson's r). Table 4 shows an intercorrelation of the subscale of the associated factors section (Pearson's r). Table 5 shows the interpretation of total score and the score of each domain in the final MCSQ. [file 9977773.f1.pdf]

Table 1. Median, interquartile range, and percentage of expert agree of each item in the third-round questionnaire (Delphi method)

Item	Median	IQR	Expert agree % (N= 19)	Note*
**Section 1. Menstrual cycle-related signs and symptoms**				
**1.1. The signs reflect the problem of the blood in the uterus**				
**The irregular menstrual blood flow**				
1. Menstrual spotting	4.55	1.13	89	Combine
2. Menstrual bleeding is less than usual or using less than 2 pads per day.	4.15	1.03	84	
3. Menstrual bleeding is heavier than usual or using more than 4 pads per day.	4.18	0.96	89	
**The irregular color of menstrual blood**				
4. Color of menstrual blood: pale red, bright red or orange	4.64	1.03	95	Combine
5. Color of menstrual blood: dark red, dark brown or black	4.64	0.98	100	
6. Many colors of menstrual blood in one period	4.77	1.25	79	
**The irregular texture of menstrual blood**				
7. Thick texture menstrual blood	4.77	1.05	84	
8. Menstrual blood looks like egg whites.	4.71	1.16	84	
**The irregular smell of menstrual blood**				
9. Menstrual blood smells stronger than usual.	4.82	0.71	100	Combine
10. Menstrual blood smells like rotten meat.	4.82	0.72	95	
**Menstrual blood clots**				
11. There are a lot of menstrual blood clots, which are released during every menstrual period or every day during the period.	4.82	0.72	95	Combine
12. Large menstrual blood clots (greater than or equal to 2 centimeters/ approximately the size of a 10-baht coin).	4.87	0.63	95	
**The irregular characteristics of leucorrhea**				
13. There is a lot of clear stretchy or thick white leucorrhea.	4.71	1.16	84	Combine
14. Need to put on a sanitary pad because there are a lot of leucorrhea.	4.55	1.06	95	
15. Leukorrhea is an abnormal color with a bad smell. (abnormal color: yellow, green, brown or blood-colored).	4.87	0.63	89	
**1.2. Menstrual cycle-related symptoms**				
**The symptoms reflect the problem of the musculoskeletal system**				
16. Skin disorder (e.g. rash, urticaria), burning sensation on skin, bruise	4.25	1.04	89	
17. Headache, dizziness	4.06	1.26	74	
18. Muscle pain, low back pain or low back pain that radiates down the legs	4.44	1.13	89	
19. Joint pain/ bone pain	4.19	1.23	79	
20. Breasts pain or tender breasts	4.11	1.13	79	
21. Abdominal cramps	4.22	1.13	84	
22. Pubic Symphysis or vaginal pain or burning sensation in vagina	4.22	1.13	84	

* Experts recommendation

Table 1. Median, interquartile range, and percentage of expert agree of each item in the third-round questionnaire (Delphi method) (continued)

Item	Median	IQR	Expert agree % (N= 19)	Note*
**1.2. Menstrual cycle-related symptoms**				
**The symptoms reflect the problem of the intestine and mesentery**				
23. Passing watery stools five or six times a day	4.00	1.46	68	
24. Nausea or vomiting	3.86	1.37	63	
25. Colic in the abdomen or flanks	3.94	1.30	68	
26. Abdominal bloating or abdominal discomfort	4.06	1.26	74	
**The symptoms reflect the problem of the gallbladder**				
27. Fatigue	3.78	1.13	63	
28. Hot and cold flushes or fever	4.55	1.23	84	
29. Waking up with fright or insomnia	3.81	1.36	63	
**The symptoms reflect the problem of heart (mind aspect)**				
30. Irritability and /or anger	4.22	1.13	84	
31. Depression and/or crying	3.86	1.42	63	
32. Anxiety and/or tension	3.86	0.14	63	
**Section 2. Associated factors**				
**2.1. Internal factors**				
**Personal data**				
33. Age	4.71	1.05	89	
34. Major elements in the human body	3.95	1.01	74	
35. Body mass index	4.09	0.89	84	
**Medical history**				
36. Underlying diseases	4.27	0.98	95	
37. Getting hit in the lower back and/ or underbelly	4.33	1.10	89	
38. Abdominal surgery	4.15	0.76	95	
**Ob-Gynecologic history**				
39. Pregnancy and childbirth history	4.18	1.18	89	
40. Postpartum care	4.45	0.99	100	
41. Abortion	4.45	0.99	100	
42. Curettage	4.27	0.98	95	
43. Contraceptive use	4.77	0.88	95	
44. Age at menarche	4.06	1.26	74	
45. Family history (grandmother/ mother/ sister/twin)	3.91	0.90	74	
46. First time menstrual cycle-related symptoms began	4.71	1.05	89	
**Behaviors**				
47. Skipping meals	4.06	1.26	74	
48. Smoking frequently	4.00	1.17	74	
49. Intense workout frequently	4.64	1.22	84	
50. Abdominal and/or hip workout frequently	4.55	1.14	89	
51. Working that requires a lot of muscle-power or energy	4.43	1.22	84	
52. Sitting for a long time during the day (more than 30 minutes/time)	3.82	1.06	68	
53. Irregular sleep schedule	4.18	0.96	89	Combine
54. Staying up late (after 10 p.m.)	4.33	1.10	89	
55. Sleep deprivation	4.71	1.16	84	

* Experts recommendation

Table 1. Median, interquartile range, and percentage of expert agree of each item in the third-round questionnaire (Delphi method) (continued)

Item	Median	IQR	Expert agree % (N= 19)	Note*
**Section 2: Associated factors**				
**Behaviors**				
56. Sleeping problem e.g. insomnia, waking up with fright in the middle night	4.22	1.13	84	
57. Constipation	4.19	1.23	79	
**Emotion**				
58. Irritability	4.71	0.97	95	
59. Anxiety and/or stress	4.71	1.05	89	
60. Sadness	4.71	1.17	84	
**2.2. External factors**				
**Types of food and drink**				
61. Eating strong-flavored food e.g. extremely spicy, extremely sour (frequently)	3.95	1.01	74	
62. Eating fried food, oily food, e.g., streaky pork, bacon, cheese (frequently)	4.06	1.29	74	Combine
63. Eating bakery, e.g., bread, cookies, cakes, pastries, and pies (frequently)	4.06	1.29	74	
64. Eating preserved food frequently	4.22	1.13	84	Combine
65. Eating uncooked food, e.g. sashimi and a medium to raw meat (frequently)	4.06	1.33	74	
66. Drinking caffeine beverages, e.g. coffee, tea, chocolate (frequently)	4.09	0.89	84	Combine
67. Drinking energy drinks frequently	3.91	0.96	74	
68. Drinking carbonated beverages frequently	3.95	0.99	74	
69. Drinking iced beverages or frappe frequently	4.27	0.98	95	
70. Drinking coconut juice frequently	4.15	0.76	95	
71. Drinking alcoholic drinks frequently	4.18	0.96	89	
**Environment**				
72. Working or staying in the area too hot (heavy sweating) for a long time	3.86	1.50	63	Combine
73. Working or staying in the area too cold (shivering) for a long time	4.21	0.90	95	
74. Working or staying in an area exposed to or inhaling chemicals frequently	4.33	1.10	89	
75. Traveling to cities or places with a different climate from the current residence frequently	4.19	1.23	79	

***** Experts recommendation

Table 2. Evaluation of the content validity of the first draft MCSQ

Item	Content validity (N = 6)		
	N[a]	I-CVI	Evaluation
**Section 1. Menstrual cycle-related signs and symptoms**			
**1.1 The signs reflect the problem of the blood in the uterus**			
1. You have light or heavy menstrual bleeding throughout the cycle (using less than 2 pads per day or more than 4 pads per day).	6	1	Excellent
2. Your menstrual blood color is pale red, bright red, orange, dark red, dark brown or black.	6	1	Excellent
3. In one period, you have many colors of menstrual blood.	6	1	Excellent
4. You have thick texture menstrual blood.	6	1	Excellent
5. Your menstrual blood looks like egg whites.	6	1	Excellent
6. Your menstrual blood smells stronger than usual or smells like rotten meat.	6	1	Excellent
7. You have a lot of menstrual blood clots which are released every day, or large size (greater than or equal to 2 centimeters).	6	1	Excellent
8. You have a lot of vaginal discharge that you need to put on a sanitary pad and your vaginal discharge is eggy, clear, stretchy or thick white.	6	1	Excellent
9. Your vaginal discharge is an abnormal color with a bad smell (abnormal color: yellow, green, brown or blood).	6	1	Excellent
10. Before the menstrual period, you have vaginal discharge and vaginal itching. (Add)	N/A	N/A	N/A
**1.2 The symptoms reflect the problem of the musculoskeletal system**			
11. Rash/ urticaria /bruise or feel burning on the skin	6	1	Excellent
12. Headache/ dizziness	6	1	Excellent
13. Muscle pain/ lower back pain	6	1	Excellent
14. Joint pain / bone pain	6	1	Excellent
15. Pubic symphysis pain or feel burning in the vagina	6	1	Excellent
16. Breast pain / tender breasts	6	1	Excellent
17. Abdominal cramps	6	1	Excellent

N[a] = Number giving rating of 3 or 4

(Add) = The item was added according to expert recommendation

N/A = item was added after evaluation of CVI that according to the comments and suggestions of the experts.

Table 2. Evaluation of the content validity of the first draft MCSQ (continued)

Item	Content validity (N = 6)		
	N[a]	I-CVI	Evaluation
**1.3. The symptoms reflect the problem of the intestine**			
18. Loose/ watery stools five or six times a day	5	0.83	Excellent
19. Nausea and/or vomiting	6	1	Excellent
20. Colic in the abdomen or flanks	5	0.83	Excellent
21. Abdominal bloating / abdominal discomfort	6	1	Excellent
**1.4. The symptoms reflect the problem of the gallbladder**			
22. Hot and cold flushes or fever	6	1	Excellent
23. Fatigue	6	1	Excellent
24. Waking up with fright / insomnia	6	1	Excellent
**1.5. The symptoms reflect the problem of heart**			
25. Irritability and /or anger	6	1	Excellent
26. Depression and/or crying	6	1	Excellent
27. Anxiety and/ or tension	6	1	Excellent
**Section 2. Associated factors**			
**2.1. Personal data**			
28. How old are you? *	6	1	Excellent
29. Date and time of birth *	6	1	Excellent
30. Weight and height *	6	1	Excellent
**2.2. Medical history**			
31. What is your health problem or underlying disease? *	5	0.83	Excellent
32. Have you ever had an accident that injured your lower back or lower abdomen? *	6	1	Excellent
33. Have you ever had abdominal surgery? *	6	1	Excellent
**2.3. Ob-Gynecologic history**			
34. How old were you when the first period start? *	6	1	Excellent
35. Have you ever given birth to a child? *	5	0.83	Excellent
36. What postpartum care did you have? *	6	1	Excellent
37. Have you ever miscarried? *	6	1	Excellent
38. Have you ever had a curettage? *	6	1	Excellent
39. Have you ever used hormonal birth control? *	6	1	Excellent
40. Has your grandmother or mother had a menstrual disorder history? *	6	1	Excellent
41. Have you ever had a curettage? *	6	1	Excellent

N[a] = Number giving rating of 3 or 4

* A multiple-choice question

Table 2. Evaluation of the content validity of the first draft MCSQ (continued)

Item	Content validity (N = 6)		
	N[a]	I-CVI	Evaluation
**2.7. The emotion and feeling**			
42. You feel anxious or worried.	6	1	Excellent
43. You are irritable or angry.	6	1	Excellent
44. You are bored, depressed, or in despair.	6	1	Excellent
**2.5. The behaviors**			
45. You eat on time and 3 meals per day.	6	1	Excellent
46. You go to bed before 10 p.m. and have deep sleep.	6	1	Excellent
47. You have sleep problems, e.g. insomnia, waking up with fright in the middle of the night.	6	1	Excellent
48. You sit for a long time during a day (more than 30 minutes/time).	6	1	Excellent
49. You work hard (using a lot of energy or muscle power).	6	1	Excellent
50. You exercise intensely.	6	1	Excellent
51. You like to train your abdomen and/or hip.	6	1	Excellent
52. You have constipation	6	1	Excellent
53. You smoke	5	0.83	Excellent
**2.4. The types of food and drink**			
54. You like to eat strong-flavored foods, e.g. extremely spicy, extremely sour.	6	1	Excellent
55. You like to eat fried food, oily food, bakery products, e.g. streaky pork, bacon, cheese, bread, cakes.	6	1	Excellent
56. You eat preserved food and/or uncooked food, e.g. fruit preserves, sashimi and medium to raw meat.	6	1	Excellent
57. You like to drink ice-beverages or frappe.	6	1	Excellent
58. You like to drink caffeine beverages (e.g. chocolate, tea, carbonated beverage, energy drinks, coffee).	6	1	Excellent
59. You like to drink coconut juice.	6	1	Excellent
60. You like to drink alcoholic drinks.	6	1	Excellent

N[a] = Number giving rating of 3 or 4, (Add) = The item was added according to expert recommendation

N/A = item was added after evaluation of CVI that according to the comments and suggestions of the experts.

Table 2. Evaluation of the content validity of the first draft MCSQ (continued)

Item	Content validity (N = 6)		
	N[a]	I-CVI	Evaluation
**2.6. The environment (hot and cool weather)**			
61. You work or stay in a bad environment for a long time a day (in the area too hot or cold / exposed to or inhaling chemicals).	6	1	Excellent
62. You travel to cities or places with a different climate from your current place of residence.	6	1	Excellent
63. In one day, you must enter and exit the area with temperature differences (hot and cold) (Add)	N/A	N/A	N/A
**S-CVI/Ave = 0.98 (61 items)**			

N[a] = Number giving rating of 3 or 4, (Add) = The item was added according to expert recommendation

N/A = item was added after evaluation of CVI that according to the comments and suggestions of the experts.

Table 3. Intercorrelation of subscale of the menstrual cycle-related signs and symptoms section (Pearson's *r*)

Subscale	1	2	3	4	5
The problem of blood in uterus	1.00				
The musculoskeletal system	0.35**	1.00			
The heart (mind aspect)	0.34**	0.62**	1.00		
The digestive system (intestine and mesentery)	0.25**	0.53**	0.44**	1.00	
Irregular menstrual blood (the impairment of blood)	0.39**	0.30**	0.22**	0.28**	1.00

** Correlation is significant at *p*-value < 0.01

Table 4. Intercorrelation of subscale of the associated factors section (Pearson's *r*)

Subscale	1	2	3	4	5
Emotion and feeling	1.00				
Type of drinks	0.18**	1.00			
Types of food	0.10*	0.23**	1.00		
Environment (hot and cold)	0.31**	0.17**	0.12**	1.00	
Behavior and health problem	0.36**	0.22**	0.02	0.22**	1.00

** Correlation is significant at *p*-value < 0.01, * Correlation is significant at *p*-value < 0.05

Table 5. The interpretation of total score and the score of each domain in the final MCSQ

Domains	No. of items	Total score	Level number (percentage)			
			None	Mild	Moderate	Severe
**1. Menstrual cycle-related signs and symptoms**	23	92	0	1-30	31-61	62-92
1.1. The problem of blood in uterus	7	28	0	1-9	10-19	20-28
1.2. Irregular menstrual blood	3	12	0	1-4	5-8	9-12
1.3. The musculoskeletal system	6	24	0	1-8	9-16	17-24
1.4. The digestive system (intestine and mesentery)	3	12	0	1-4	5-8	9-12
1.5. The heart (mind aspect)	4	16	0	1-5	6-11	12-16
			**Very good**	**Good**	**Fair**	**Bad**
**2. Associated factors**	12	48	0	1-16	17-32	33-48
2.1. Emotion and feeling	3	12	0	1-4	5-8	9-12
2.2. Types of drinks	2	8	0	1-2	3-5	6-8
2.3. Types of food	2	8	0	1-2	3-5	6-8
2.4. Environment	2	8	0	1-2	3-5	6-8
2.5. Behaviors and health problems	3	12	0	1-4	5-8	9-12

The researcher set the criteria for interpreting results of each section by dividing the score into 4 levels.
